# Comparative Evaluation of the Effects of Calcium Hydroxide Intracanal Medicament and Antibiotic Paste on Dentin Microhardness: A Systematic Review

**DOI:** 10.7759/cureus.78886

**Published:** 2025-02-12

**Authors:** Indumathi Manoharan, Pradeeba Anandi Jeya Goutham, Muralidasan Kalaivani, Harini Madhavan, Dakshayani Balaji, Shanthi K, Sandhya Shanmugam

**Affiliations:** 1 Conservative Dentistry and Endodontics, Sri Ramachandra Dental College and Hospital, Sri Ramachandra Institute of Higher Education and Research (SRIHER), Chennai, IND; 2 Conservative Dentistry and Endodontics, Sree Balaji Dental College and Hospital, Bharath Institute of Higher Education and Research, Chennai, IND; 3 Public Health Dentistry, Sri Ramachandra Dental College and Hospital, Sri Ramachandra Institute of Higher Education and Research (SRIHER), Chennai, IND; 4 Conservative Dentistry and Endodontics, DA Pandu RV Dental College and Hospital, Bangalore, IND

**Keywords:** calcium hydroxide paste, double antibiotic paste, endodontic regeneration, intracanal medicament, microhardness, modified triple antibiotic paste, triple antibiotic paste

## Abstract

An infectious environment is reported to hinder the process of pulp tissue regeneration by damaging the cells responsible for tissue formation, including stem cells in the periapical tissues. Therefore, a sterile environment is critical for pulp tissue regeneration, which can be achieved by copious irrigation and intracanal medicament (ICM) placement. This systematic review compares the effect of calcium hydroxide (Ca(OH)_2_) and various antibiotic pastes, such as triple antibiotic paste (TAP), modified triple antibiotic paste (MTAP), and double antibiotic paste (DAP),* *on dentin microhardness when used as ICMs.

MeSH terms and specific keywords were used to search Scopus, EBSCOhost, Cochrane Library, and PubMed. In vitro studies that compared the impact of Ca(OH)_2_ and antibiotic pastes, such as TAP, MTAP, and DAP,* *on dentin microhardness were included. Data from the included articles were extracted, and their quality was assessed using the revised Quality Assessment Tool for In Vitro Studies (QUIN) tool. Seven articles were included for the qualitative synthesis, and data were extracted from each. Within the scope of this systematic review, Ca(OH)_2_, an alternative ICM recommended by the American Association of Endodontics* *Glossary of Endodontic Terms for REPs, showed a lesser reduction in microhardness than TAP. DAP and MTAP caused a reduction in dentin microhardness compared to that of Ca(OH)_2_.

## Introduction and background

Root canal treatment of immature necrotic teeth has indeed posed a significant challenge in clinical practice due to the unique anatomical and biological properties of these teeth. Immature necrotic teeth have fragile roots with thin dentin walls, and wide apical foramens, making root canal sealing and apical closure challenging [1]. Traditionally, these cases were managed using calcium hydroxide (Ca(OH)_2_) apexification, a technique designed to promote the calcific barrier formation at the apical foramen, thereby achieving apical closure without actual root development [2]. This required prolonged use of Ca(OH)_2_, which is linked to a higher risk of cervical fracture in immature permanent teeth [3]. Later the apexification technique was practiced using mineral trioxide aggregate (MTA) [4], which facilitated single-step apexification, eliminating the need for multiple appointments and reducing the risk of fracture [5]. While apexification with Ca(OH)_2_ or other bioceramic materials has been widely employed, it is crucial to note that this treatment option does not promote root maturation or dentin wall thickening, thereby increasing the risk for fracture [6].

Later a significant shift occurred toward more dynamic management that aimed to regenerate the dentin-pulp complex rather than simply sealing the root end [7]. This approach, referred to as regenerative endodontic procedures (REP), aims to not only achieve apical closure but also promote root growth and increase dentinal wall thickness, leading to improved long-term outcomes [8]. This technique employs a triad comprising stem cells, growth factors, and biomimetic scaffolds [9]. Establishing a sterile environment within the root canal using intracanal antibacterial medicaments is a key factor for the success of endodontic regeneration protocol [10,11]. The presence of infection is reported to affect the stem cells essential for regenerative endodontics, leading to failure of the REPs [12].

The American Association of Endodontists (AAE) outlines a clinical protocol for REPs. This includes disinfecting the canal with 20 ml of 1.5% sodium hypochlorite irrigating solution per canal for five minutes on the first visit, followed by intracanal medicament (ICM) placement for one to four weeks to further eliminate any remaining microorganisms. Once the symptoms have resolved, the canal is irrigated with 20 ml of 17% EDTA solution in the second visit, after which bleeding is induced to form a blood clot inside the canal [13].

Ca(OH)_2_ has been the gold standard ICM for years due to its high pH, which neutralizes bacterial byproducts and provides antimicrobial activity [14]. Additionally, Ca(OH)_2_ is biocompatible and aids in the viability of the stem cells from the apical papilla (SCAPs) and its proliferation, which are crucial for pulp regeneration [15]. However, long-term exposure can disrupt the radicular dentin’s chemical structure, weakening the root's mechanical integrity. This is particularly concerning in immature teeth, where dentin integrity is already compromised [10,16,17].

Triple antibiotic paste (TAP) containing ciprofloxacin, metronidazole, and minocycline was demonstrated to have effectively decontaminated *Escherichia coli*‑infected dentin. The limitations of TAP include discoloration of immature teeth, likely caused by minocycline, and higher concentrations may affect stem cell vitality [18,19]. To prevent discoloration from minocycline, double antibiotic paste (DAP), made of metronidazole and ciprofloxacin, and modified triple antibiotic paste (MTAP) where minocycline in TAP is substituted with clindamycin has been suggested and successfully used in regeneration procedures [19,20]. However, antibiotic pastes are known to be acidic in nature [21]. Therefore, prolonged exposure of root dentin to it must be properly researched as acids have a demineralizing effect on dentin.

Although numerous studies have analyzed the effect of antibiotic pastes with Ca(OH)_2_ on the microhardness of dentin, the results are inconsistent. Furthermore, clinical evidence on this issue is limited. This systematic review aimed to compare the effect of TAP, DAP, and MTAP with Ca(OH)_2_ on dentin microhardness from the available ex vivo studies.

## Review

Materials and methods

A protocol was readied and listed in the Open Science Framework (OSF) database (https://doi.org/10.17605/OSF.IO/PGKRQ), an international prospective registry of systematic reviews before initiation of the search for articles. The review was then conducted following the Preferred Reporting for Systematic Review and Meta-Analysis (PRISMA) guidelines.

Inclusion Criteria

This systematic review included (1) all in vitro studies focusing on the effect of any one or more antibiotic ICM and Ca(OH)_2_ on the microhardness of dentin in extracted human permanent teeth, (2) articles published in English-language with full-text availability, and (3) publications between 2000 and 2024.

PICO Question

This systematic review used the Population, Intervention, Comparison, Outcome, and Study (PICOS) framework to develop the research question which is “Does the type of ICM used in REPs affect the microhardness of dentin when assessed in in vitro studies?” (P = Dentin samples of extracted human permanent teeth; I = antibiotic pastes including TAP, DAP, and MTAP; C = Ca(OH)_2_; O = microhardness; and S = in vitro studies).

Exclusion Criteria

This systematic review excluded the in-vivo studies, in vitro studies that were conducted using deciduous teeth, animal studies, and studies in languages other than English.

Methods of Search

Eligible studies were typically identified through extensive electronic searches across four databases from January 2000 to July 2024. The Cochrane Library, Scopus database, PubMed database, and EBSCO host were searched. To find new, relevant studies, reference lists from identified in vitro studies and review papers were manually examined. Reading the entire text was also used to evaluate potentially eligible studies, and the final decision regarding addition was made. The search terms were (((((((((((((((((((regenerative) OR (endodontics)) OR (regenerative endodontic)) OR (regenerative endodontic procedure)) OR (immature teeth)) OR (open apex)) OR (necrotic immature teeth)) OR (permanent immature teeth)) OR (REP)) OR (pulp regeneration)) OR (revascularization)) OR (revitalization)) OR (root dentin)) OR (human dentin)) OR (dentin)) OR (radicular dentin)) OR (coronal dentin))) AND ((((((((((((antibiotic intracanal medicaments) OR (antibiotic pastes)) OR (triple antibiotic paste)) OR (TAP)) OR (double antibiotic paste)) OR (DAP)) OR (Modified triple antibiotic paste)) OR (MTAP)) OR (minocycline)) OR (ciprofloxacin)) OR (metronidazole)) OR (clindamycin))) AND (((((Calcium hydroxide) OR (Ca(OH)2)) OR (Intracanal medicament)) OR (root canal medicament)) OR (Calcium hydroxide intracanal medicament))) AND (((((((microhardness) OR (hardness)) OR (vicker's microhardness)) OR (knoop's microhardness)) OR (root dentin microhardness)) OR (radicular dentin microhardness)) OR (dentin microhardness)).

Study Selection

The complete list of articles was independently reviewed by two researchers, who selected the potentially applicable articles first by title and then by abstract. After that, the articles meeting the inclusion criteria were separated by full‑text screening. In the event of disagreement, arguments were debated until agreement was reached, or a third author with additional experience was consulted to reach a final agreement.

Extraction of Data

The following data was extracted from each involved article: name of the primary author, year of publication, sample size, type of human extracted permanent tooth used, ICM placed, type of test employed to measure microhardness, outcome obtained, and conclusion.

Quality Assessment

The “Quality Assessment Tool for In Vitro Studies” (QUIN tool) was utilized to assess and evaluate the risk of bias of the included in vitro studies in this systematic review [22]. The QUIN tool consists of 12 criteria, which are scored as adequately specified (score = 2), not adequately specified (score = 1), not specified (score = 0), or not applicable. The total scores (TS) from the 12 criteria are obtained, and the following formula is used to calculate the final score (FS) percentage: FS% = (TS × 100)/(2 × count of applicable criteria). According to this percentage, each study is classified as high, medium, or low when the FS% is >70%, 50%-70%, and <50%, respectively.

Results

Extraction of Data

**Figure 1:**
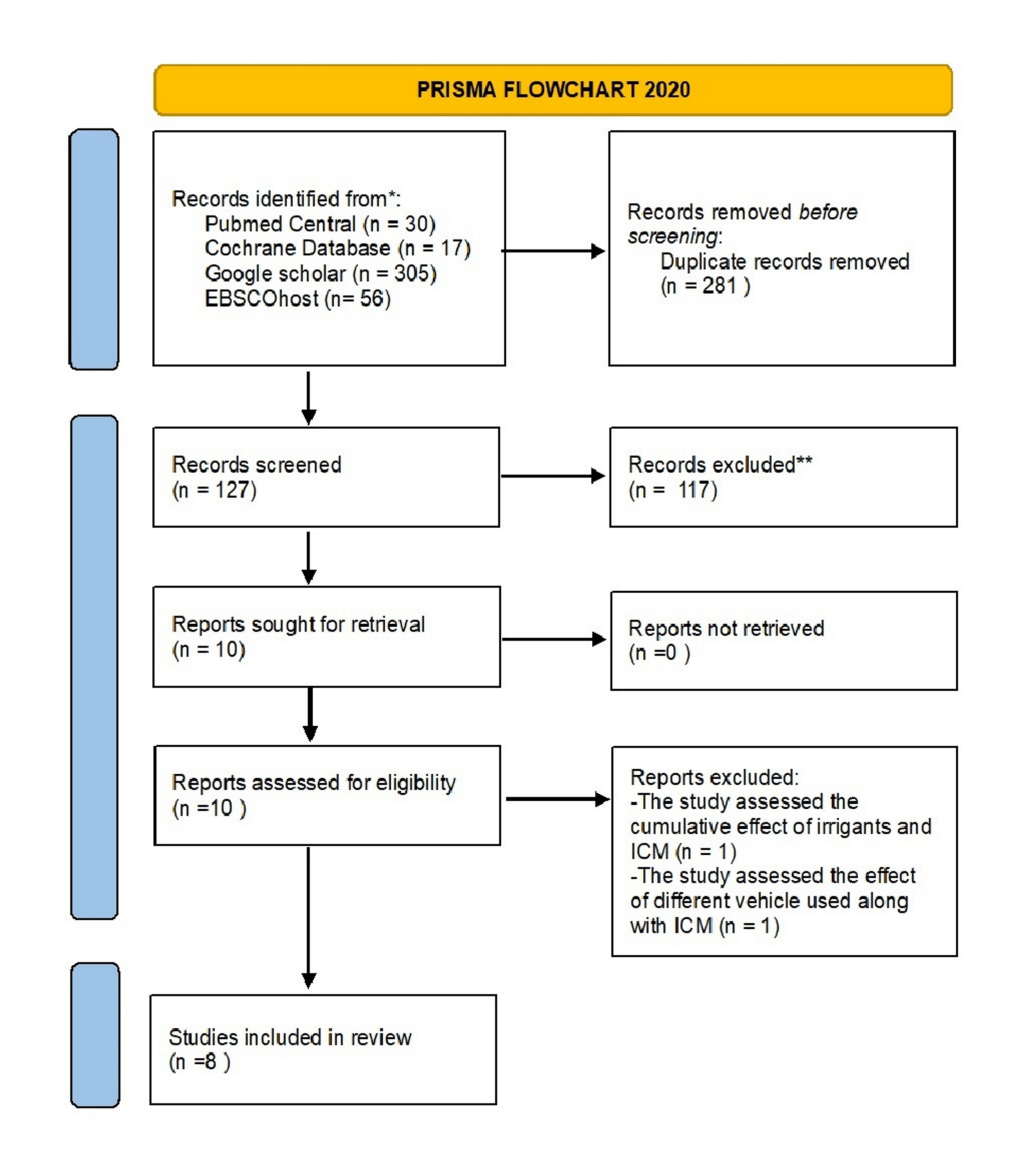
PRISMA study flow chart depicting the article screening and inclusion procedure *ICM: Intracanal medicament.

The article inclusion procedure is depicted in the PRISMA study flow diagram in Figure 1. Initially, 408 records were screened. The duplicates were removed, and studies were excluded based on title and abstract review. Ten studies remained for full-text examination. Eight studies [19,23-29] meeting the inclusion criteria were taken up for qualitative assessment. Two studies did not follow the inclusion criteria and were excluded. One of the excluded studies assessed the cumulative effect of different root canal irrigants along with ICM [30], and the other study assessed the effect of different vehicles used along with the ICM on the microhardness of dentin [17].

Characteristics of Included Studies

Between 2013 and 2024, the eight included studies had a total of 807 samples. These samples were roots of maxillary incisors, maxillary and mandibular premolars, and maxillary and mandibular third molars. The extracted data are tabulated in Table 1.

**Table 1:** Study characteristics of the included studies in this systematic review *TAP: Triple antibiotic paste; DAP: Double antibiotic paste; MTAP: Modified triple antibiotic paste; Ca(OH)_2_: Calcium hydroxide; ICM: Intracanal medicament.

Author name and year	Total sample size	Tooth used	ICM placed	Microhardness test employed	Outcomes	Inference
Yassen et al., 2015 [19]	N = 50	Extracted third molars (coronal dentin)	Untreated (control 1, control 2) 1 g/ml TAP, 1 mg/ml TAP, Ca(OH)_2_	Vickers microhardness test	After four weeks, dentin treated with 1 gm/ml TAP had significantly lesser microhardness values compared to Ca(OH)_2_ and diluted TAP (1 mg/1 ml) (p < 0.0001).	TAP at higher concentrations (1 g/1 ml) caused a significant reduction in the microhardness of dentin than Ca(OH)_2_.
Amonkar et al., 2021 [23]	N = 180	Extracted single-rooted premolars (radicular dentin)	TAP, Ca(OH)_2_, Ledermix	Vickers microhardness	Dentin treated with Ca(OH)_2_ significantly decreased the microhardness after 1 week, 4 weeks, and 12 weeks compared to TAP (p < 0.0001) at 500 µm from pulp space.	Ca(OH)_2_ significantly decreases the microhardness of root dentin compared to TAP in the short and long terms.
Chandak et al., 2020 [24]	N = 45	Extracted third molars (radicular dentin)	Untreated (control) MTAP, and Ca(OH)_2_	Vickers microhardness	After one week, there was no significant difference in microhardness of radicular dentin between MTAP and Ca(OH)_2_ at 1000 µm from pulp space (p > 0.05).	The reduction in root detin microhardness caused by placing MTAP for one week was comparable to Ca(OH)_2_.
Hamdy et al., 2020 [25]	N = 168	Extracted maxillary central incisor (radicular dentin)	Untreated (control), bioactive glass, DAP, and Ca(OH)_2_	Vickers microhardness	Ca(OH)_2 _significantly decreased the microhardness of radicular dentin after 4 and 12 weeks compared to DAP (p < 0.0001) at 500 µm from pulp space.	Long-term use of Ca(OH)_2 _ICM decreases the microhardness of root dentin compared to DAP.
Yassen et al., 2013 [26]	N = 180	Extracted mandibular single-rooted premolars (radicular dentin)	Untreated (control), Ca(OH)_2_, TAP, and DAP	Knoop microhardness	Dentin treated with TAP and DAP significantly decreased the microhardness of root dentin than Ca(OH)_2_ (p < 0.0001 at 500 µm and p < 0.0005 at 1000 µm from pulp space) after 4 and after 12 weeks (p < 0.0001 at both depths).	TAP and DAP induced a decrease in microhardness of radicular dentin after one month and above compared to Ca(OH)_2_.
Parashar et al., 2015 [27]	N = 50	Extracted mandibular single-rooted premolars (radicular dentin)	Untreated (control), Aloe vera, MTAP, and Ca(OH)_2_	Knoop microhardness	After two weeks, there was no significant difference in the microhardness of radicular dentin between MTAP and Ca(OH)_2_ at 1000 µm from the pulp space (p > 0.05).	The reduction in root detin microhardness caused by placing MTAP for two weeks was comparable to Ca(OH)_2_.
Yilmaz et al., 2015 [28]	N = 70	Extracted maxillary incisors (radicular dentin)	Untreated (control), TAP, DAP, and Ca(OH)_2_	Knoop microhardness	Dentin treated with TAP and DAP showed a significant decrease in microhardness after 1 week, 2 weeks, and 4 weeks compared to Ca(OH)_2 _(p < 0.0001).	Applying TAP and DAP caused a significant reduction of root dentin microhardness compared to Ca(OH)_2_ in the short and long terms.
Nogueira et al., 2024 [29]	N = 64	Extracted mandibular single-rooted premolars (radicular dentin)	Untreated (Control), Ca(OH)_2_, TAP, and DAP	Knoop microhardness	Ca(OH)_2_ group showed significantly higher microhardness of dentin than TAP and DAP, after 20 days at 25, 50, and 100 μm from the canal lumen (p < 0.05).	TAP and DAP caused a significant reduction in root dentin microhardness than Ca(OH)_2_ at the end of 20 days.

Intervention

The samples of the included invitro studies had compared the dentin microhardness following placement of antibiotic paste as ICM with Ca(OH)_2_. Dentin microhardness was evaluated using Vicker’s microhardness test [19,23-25] and Knoop’s microhardness test [26-29]. Based on the criteria of each study, the microhardness values were evaluated in samples prepared from radicular dentin [23-29]. In the Yassen et al. study, a coronal disc of dentin from the third molar was used for the microhardness test post-exposure to ICM [19].

Outcomes

Out of five studies that compared the TAP with Ca(OH)_2_, four studies reported significantly more reduction in microhardness in the TAP group compared to Ca(OH)_2_ group [19,27-29], while one study reported a contrary result [23]. Two studies compared the effect of MTAP with Ca(OH)_2_ on dentin microhardness and reported no significant differences among the groups [24,27]. Among three studies that studied the influence of Ca(OH)_2_ and DAP on microhardness, two reported DAP to have significantly more reduction in microhardness than the Ca(OH)_2_ group [28-30]. On the contrary, the other study reported that the Ca(OH)_2_ group caused more reduction in microhardness compared to the DAP group [25].

Quality Assessment

Estimates for the risk of bias categories are shown in Table 2. All eight studies were assessed to have moderate risk. The overall risk of bias is moderate. Conflict of interest was denied in all the included studies. None of the included studies reported to have financial conflict of interest.

**Table 2:** Quality assessment of the included invitro studies in this systematic review

Criteria	Yassen et al., 2015 [19]	Amonkar et al., 2021 [23]	Chandak et al., 2020 [24]	Hamdy et al., 2020 [25]	Yassen et al., 2013 [26]	Parashar et al., 2020 [27]	Yilmaz et al., 2015 [28]	Nogueira et al., 2024 [29]
Clearly stated aims/objectives	2	2	2	2	2	2	2	2
Detailed explanation of the sample size calculation	0	0	0	0	0	0	0	2
Detailed explanation of the sampling technique	2	2	2	2	2	2	2	2
Details of the comparison group	2	2	2	2	2	2	2	2
A detailed explanation of the methodology	2	2	2	2	2	2	2	2
Operator details	0	0	0	0	0	0	0	0
Randomization	0	0	0	0	0	0	0	0
Method of measurement of outcome	2	2	2	2	2	2	2	2
Outcome assessor details	0	0	0	0	0	0	0	0
Blinding	0	0	0	0	0	0	0	0

Discussion

A systematic review by Wikström et al. in 2021 concluded that regenerative procedures had survival and success rates equal to those of apexification, proving effective in treating immature necrotic permanent teeth. Additionally, endodontic regenerative techniques compared to apexification techniques showed more desirable outcomes such as root wall thickening and root lengthening [31]. While root wall thickness increased mainly in the mid- and apical regions, the cervical area showed minimal to no changes, leaving it more susceptible to fracture in immature necrotic teeth [5,6,32].

In regenerative endodontic treatments, mechanical preparation is skipped or kept minimal to preserve the weak roots of immature permanent teeth, focusing instead on chemical preparation using irrigation solutions and medicaments [33]. In endodontic regeneration, ICM duration ranges from one to 11 weeks, making it crucial to assess their effects on root dentin over time [34].

Microhardness testing indirectly measures the loss or gain of mineral content in dental hard tissues, reflecting the calcified matrix per square millimeter [35]. Prabhakar et al. noted it as the most practical method for quantifying demineralization and assessing dentin's interaction with various medicaments [36]. Also, measurements of hardness can be correlated with mechanical properties such as fracture resistance, yield strength, and modulus of elasticity [37].

Ca(OH)_2_ is a selected ICM in this systematic review, as it is recommended by the AAE as an alternative for RET [13]. It does not stain teeth and stimulates the growth factors released from dentin. However, the highly alkaline pH of Ca(OH)_2_ is neutralized by the buffering effect of dentinal fluid, allowing *E. faecalis* survival, which can compromise REP success [38]. Therefore, the need for an ICM that is effective in eradicating *E. faecalis* such as antibiotic ICM must be considered.

TAP and Ca(OH)_2_

Out of eight, four studies have reported a significantly higher decrease in dentin microhardness caused by TAP than Ca(OH)_2_ [19,26,28,29]. Only one study reported a higher reduction in dentin microhardness by Ca(OH)_2_ [23].

DAP and Ca(OH)_2_

Out of eight studies, three have reported a significantly higher reduction of microhardness caused by DAP compared to Ca(OH)_2_ [26,28,29]. One study reported a higher reduction in dentin microhardness by Ca(OH)_2_ [25].

MTAP and Ca(OH)_2_

Two out of eight studies have studied the impact of MTAP with Ca(OH)_2_ on root dentin microhardness. Both reported MTAP to have a reduction in dentin microhardness that was comparable to that of Ca(OH)_2_ [24,27].

Though it was concluded that TAP is the most effective ICM against *faecalis*, its effect on root dentin should also be considered before use [39]. In 2013, Yassen et al. studied the impact of ICMs on the root dentin’s chemical structure in human immature teeth using ATR-FTIR spectroscopy to analyze the phosphate-to-amide ratio. They found that TAP, with an acidic pH of 2.9, reduced this ratio more than other ICMs including DAP and Ca(OH)_2_, leading to greater demineralization [40]. Minocycline in TAP was noted for its calcium-chelating properties, contributing to dentin demineralization [41]. This aligns with Gúzman et al.'s 2022 study [30]. Both studies showed that this demineralization of inorganic content reduced the microhardness of exposed dentin.

Acids are incorporated into antibiotics for chemical stabilization, tonicity regulation, and physiological compatibility. The pH of DAP is 3.4, which may explain why DAP reduces microhardness in the majority of studies like TAP when compared to Ca(OH)_2_ [40]. The slightly higher pH of MTAP and the absence of minocycline, a calcium-chelating agent, makes MTAP an alternative ICM with a lesser demineralizing effect, which is comparable to Ca(OH)_2_ [41].

While antibiotic ICMs affect the inorganic content, Ca(OH)_2_ impacts the organic content through protein denaturation or breakdown due to its high pH [40]. As collagen contributes to the toughness of hard tissues, a degraded collagen matrix leads to more mineralized dentin with increased brittleness and reduced toughness [42]. It was found that Ca(OH)_2_ particle size correlates with that of the dentinal tubule, allowing it to directly penetrate the apatite-encapsulated collagen matrix. This alters tropocollagen’s 3D structure, which decreases the elastic modulus and microhardness of dentin [43,44].

The results of this systematic review should be inferred cautiously due to the moderate risk of bias of included articles and the inability to perform a meta-analysis due to heterogeneities of the extracted data. A meta-analysis was not conducted due to the heterogeneity observed among the included studies, including: (i) variations in the types of teeth used, (ii) differences in the types of dentin samples, (iii) microhardness measurements taken at different levels from the canal lumen in each study, and (iv) variations in the testing methods used to measure microhardness. A recent review by Vatankhah et al. found that single antibiotic pastes (SAP) used as ICM showed antibacterial efficacy comparable to TAP and superior to DAP and Ca(OH)_2_ [39]. However, the effect of SAP on dentin's mechanical properties has not been extensively studied. Further standardized studies are needed to achieve more consistent and reliable results.

## Conclusions

Within the scope of this systematic review, it can be concluded that TAP results with the highest reduction of dentin microhardness among other ICMs followed by DAP. Ca(OH)_2_, an alternative ICM recommended by AAE, shows a lesser reduction in microhardness than TAP and DAP. The MTAP caused a reduction in the microhardness of dentin comparable to that of Ca(OH)_2_. Additionally, it overcomes the drawbacks of TAP, such as staining of the teeth, and therefore can be an alternative antibiotic ICM in REP.
